# Beyond Traditional Risk Factors: Cocaine Abuse and Pulmonary Embolism

**DOI:** 10.7759/cureus.80915

**Published:** 2025-03-20

**Authors:** Guillermo Loyola, Oscar Diaz, Kevin Sande, Fernando Carreras Calvo, Muhammad Farooq

**Affiliations:** 1 Internal Medicine, University of Florida College of Medicine – Jacksonville, Jacksonville, USA; 2 Dr. Kiran C. Patel College of Osteopathic Medicine, Nova Southeastern University, Fort Lauderdale, USA; 3 Internal Medicine, Palmetto General Hospital, Miami, USA

**Keywords:** cocaine abuse, cocaine-induced pulmonary embolism, d-dimer, pulmonary embolism, pulmonary thrombectomy

## Abstract

Pulmonary embolism (PE) is a critical and potentially life-threatening condition necessitating prompt diagnosis and intervention due to its significant risk of morbidity and mortality. We present a case of a 42-year-old male with a history of cocaine abuse who was admitted with a massive PE (high risk), defined by hemodynamic instability or evidence of significant right ventricular dysfunction. In this case, the severity was suggested by syncope, right heart strain, and elevated cardiac biomarkers. The patient reported sudden onset shortness of breath, sharp chest pain exacerbated by inspiration, and a syncopal episode. Clinical evaluation revealed tachycardia and hypoxemia. Diagnostic tests showed elevated D-dimer levels and signs of right ventricular strain on echocardiography. Computed tomography pulmonary angiography (CTPA) confirmed acute bilateral pulmonary emboli, with a large clot burden predominantly in the right pulmonary artery. The patient underwent a mechanical thrombectomy, which significantly reduced the clot burden and improved pulmonary artery perfusion.

## Introduction

Pulmonary embolism (PE) is a potentially fatal disorder characterized by obstruction of a pulmonary artery or its branches. The condition might be of great illness and can even cause death; thus, it calls for immediate diagnosis and management. The intricate relationship that exists between the abuse of substances and thromboembolic events has been a big issue in clinical medicine, which calls for full exploration and clinical address.

Cocaine is a potent vasoactive drug, associated with arterial and venous thrombotic events. This drug is known to have prothrombotic properties and direct effects on the blood vessel intima [1]. Moreover, cocaine use is associated with tachycardia and marked acute elevations of blood pressure due to inhibition of catecholamine reuptake at presynaptic nerve terminals. This accentuates the thromboembolic risk [2].

PE, on the other hand, is a serious type of thromboembolic event, where a blood clot travels through the venous system and lodges in the pulmonary arteries. This blocks blood flow to parts of the lung, which can impair oxygen exchange and increase strain on the right side of the heart. In severe cases, this may lead to life-threatening consequences such as significant hypoxemia, arrhythmias, or shock. Thromboembolic events encompass both arterial and venous clot formations, but a PE specifically refers to clots within the pulmonary vasculature. PE can result from provoked or unprovoked events, with provoked events occurring after triggering factors in the previous three months, such as immobilization, trauma, surgery, cancer, or hospitalization [3]. Early recognition and treatment, such as anticoagulation, are crucial in preventing further complications.

We present a case of a 42-year-old male with a history of cocaine abuse who was admitted with a near-fatal PE. He complained of sudden difficulty in breathing, with sharp chest pain that was worse with breathing. He was noted to have an abnormally fast heartbeat and low oxygen levels in his blood. His diagnosis was confirmed to be a PE through computed tomography pulmonary angiography (CTPA), demonstrating a significant embolus in the right pulmonary artery. Laboratory studies revealed an elevated D-dimer level and right ventricular strain on echocardiography, which are truly indicative of the severity of the embolic event this patient suffered.

## Case presentation

**Figure 1:**
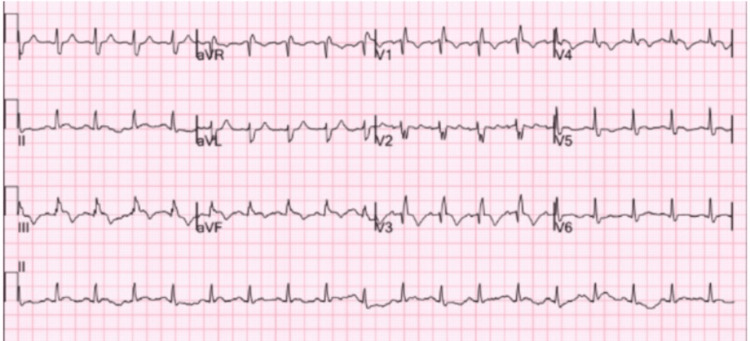
Electrocardiogram showing sinus tachycardia with T-wave inversions in leads II, III, aVF, and V1-V4, indicative of right ventricular strain. aVF: augmented vector foot.

A 42-year-old male with a past medical history significant for chronic cocaine use, with his last known use occurring within 24 hours prior to presentation, presented after experiencing a syncopal event. He described the episode as a sudden loss of consciousness while standing, followed by immediate recovery upon hitting the floor. There was no reported history of other chronic medical conditions such as hypertension, diabetes, or prior thromboembolic events. He described supporting a three-day record of deteriorating shortness of breath and dizziness. The patient was found to be in mild respiratory distress, with an oxygen saturation of 88% on room air. His vital signs revealed a heart rate of 122 beats per minute and a blood pressure of 88/56 mmHg, consistent with hemodynamic instability. He appeared tachycardic on examination, with elevated jugular venous pressure and clear lung fields. The ECG, depicted in Figure 1, demonstrated sinus tachycardia with T-wave inversions in the inferior leads (II, III, augmented vector foot (aVF)) and anterior precordial leads (V1-V4), consistent with right ventricular strain. The laboratories revealed an elevated troponin and pro-B-type natriuretic peptide (pro-BNP).

**Figure 2:**
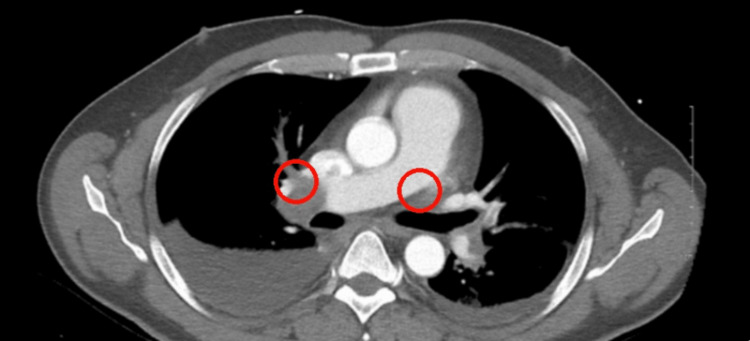
CT scan showing bilateral pulmonary embolisms.

**Figure 3:**
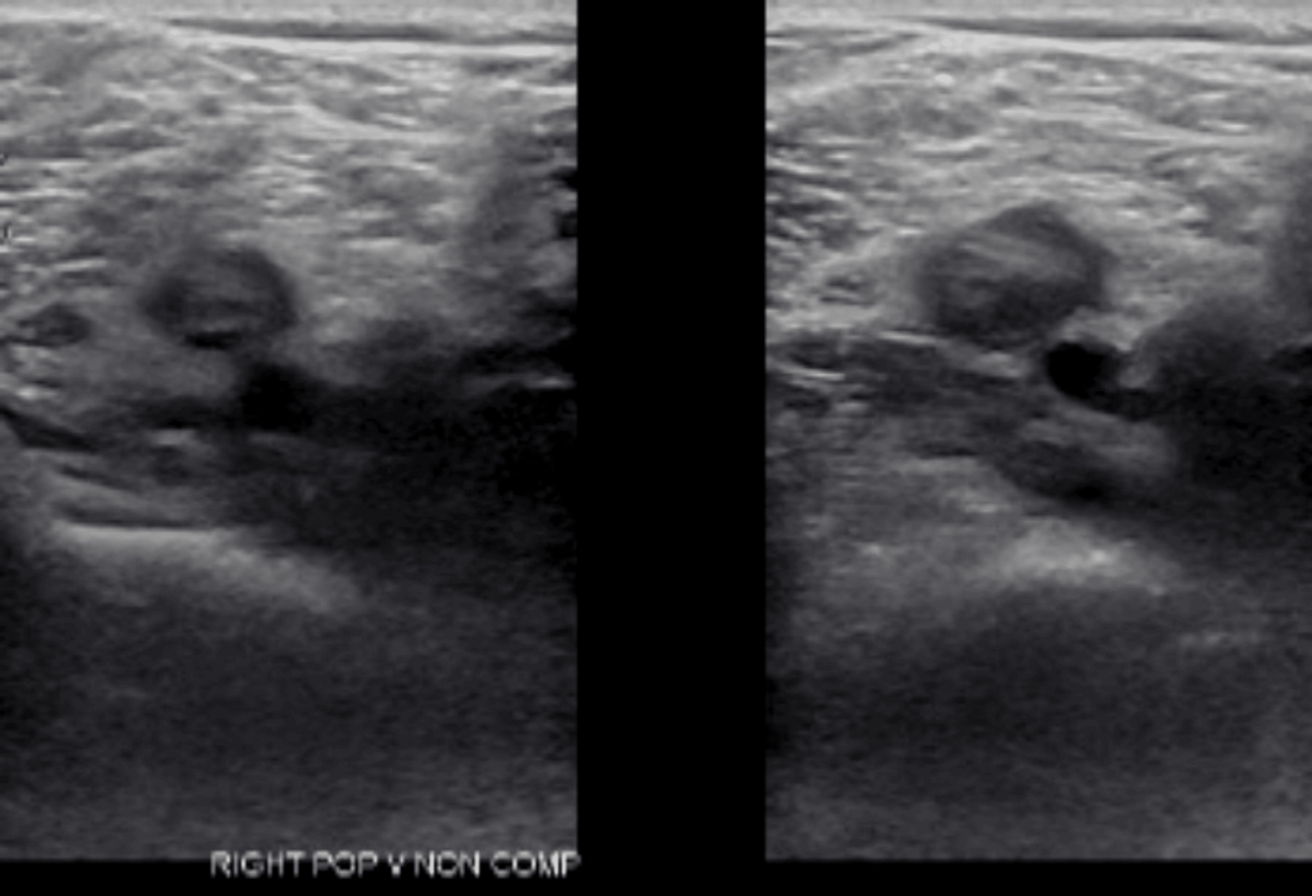
Doppler ultrasound showing deep venous thrombosis of the right popliteal vein.

A ventilation/perfusion lung scan was ordered that revealed a high probability of a PE, further backed by elevated D-dimer levels as depicted in Table 1. A CT angiography of the chest was done, showing acute bilateral pulmonary emboli, with a larger embolus burden observed in the right pulmonary artery, as shown in Figure 2. There was evidence of right heart strain with a ratio of 1.35 between the right ventricle and left ventricle size ratio. Doppler ultrasound of the lower extremity revealed deep vein thrombosis in the right popliteal vein, as shown in Figure 3. The interventional cardiology team evaluated the patient and performed a mechanical thrombectomy for removal of the pulmonary emboli. Before the intervention, the mean pulmonary artery pressure was 47 mmHg (normal: 9-18 mmHg), and the pulmonary capillary wedge pressure was 23 mmHg (normal: 6-12 mmHg) on right heart catheterization. These elevated pressures are consistent with significant pulmonary hypertension secondary to the pulmonary embolism. The clot burden was significantly decreased by more than 90% with remarkable improvement in filling of all pulmonary arteries during the venous phase. Symptomatically, he improved after the operation.

**Table 1:** Abnormal lab values obtained upon admission. Key abnormalities are seen in the patient's D-dimer level, troponin I levels, and urine cocaine screen.

Abnormal lab values	Patient’s lab value	Reference value
Hematocrit	51.2%	42.0-52.0%
Mean corpuscular volume	93.4 fL	80-94 fL
D-dimer, quantitative	5.96 mg/L FEU	0.00-0.49 mg/L FEU
Potassium	5.3 mmol/L	3.4-5.0 mmol/L
Carbon dioxide	19 mmol/L	22-30 mmol/L
Creatinine	1.30 mg/dL	0.66-1.25 mg/dL
Estimated glomerular filtration rate	70 ml/min/1.73m2	>90 ml/min/1.73m2
Glucose	162 mg/dL	74.0-106.0 mg/dL
Troponin I	0.361 ng/mL	0.012=0.034 ng/mL
Urine cocaine screen	Positive	Negative

## Discussion

Many of the prothrombotic effects of cocaine involve activation of the coagulation process and stimulate the production of von Willebrand factor [4]. Von Willebrand factor combines with other prothrombotic agents in the coagulation cascade to finally form blood clots. The impurities in cocaine, including levamisole, complicate its action within the body, increasing damage to and scarring of the lungs and enhancing the risk of blood clotting-related complications. Levamisole is a common adulterant in cocaine and an anthelmintic drug associated with agranulocytosis and vasculitis [5]. Because of this, cocaine can induce direct damage to endothelial cells, stimulate inflammation, and thereby aggravate its prothrombotic effect. The case above describes that suspicion of cocaine-induced pulmonary embolism has to be extremely high in the differential diagnosis for patients having substance abuse histories with symptoms characteristic of thromboembolic events. Doctors should maintain a high index of suspicion for symptoms and signs of PE, including acute dyspnea, pleuritic chest pain worsened by inspiration, and hemoptysis, particularly in individuals with known or suspected cocaine use [6]. It is also important to note that diagnostic modalities such as D-dimer tests, echocardiography, and CTPA are essential for establishing the diagnosis as accurately as possible and ensuring timely intervention.

The management of cocaine-induced pulmonary embolism requires an interdisciplinary approach. It should involve early anticoagulation, supportive care, and treatment for the underlying substance use disorder.

Anticoagulation is the cornerstone of therapy for pulmonary embolism. Initial options include unfractionated heparin (UFH), which is often preferred in unstable patients or those undergoing interventions such as thrombolysis or thrombectomy due to its rapid onset and reversibility. Low molecular weight heparin (LMWH), such as enoxaparin, is commonly used in stable patients and facilitates outpatient management. Direct oral anticoagulants (DOACs), including rivaroxaban and apixaban, are increasingly used due to their ease of administration and do not require laboratory monitoring. Vitamin K antagonists like warfarin are also an option but require frequent international normalized ratio (INR) monitoring and dietary restrictions. The choice of anticoagulant depends on the patient’s clinical status, comorbidities, renal function, and risk of bleeding.

In this case, the patient underwent mechanical thrombectomy and was initiated on anticoagulation therapy post procedure.

Still, the reduction of cocaine consumption with public health interventions and the dissemination of education regarding the dangers of adulterated drug use are important preventive measures. This case calls attention to the broader implications of substance use disorders for cardiovascular health and highlights the critical role of healthcare professionals in reducing these risks through early detection and effective intervention.

## Conclusions

Pulmonary embolism is a serious, potentially fatal condition that requires prompt diagnosis and management. This case highlights the complex relationship between substance abuse, particularly cocaine, and thromboembolic events. Cocaine's prothrombotic properties and its impact on blood vessels significantly increase the risk of pulmonary embolism, as illustrated by the severe case of a 42-year-old male with a history of cocaine abuse. His presentation with acute dyspnea, chest pain, and syncope, alongside diagnostic findings of elevated D-dimer, right ventricular strain, and significant emboli on CT angiography, underscores the need for high clinical suspicion and thorough evaluation in similar patients. The interdisciplinary management approach, including mechanical thrombectomy and anticoagulation, proved effective in this case. This emphasizes the importance of early intervention and the critical role healthcare professionals play in mitigating risks associated with substance use disorders, accentuating the need for preventive public health measures and education.
